# Biocompatibility and Stability of Polysaccharide Polyelectrolyte Complexes Aimed at Respiratory Delivery

**DOI:** 10.3390/ma8095268

**Published:** 2015-08-28

**Authors:** Susana Rodrigues, Lurdes Cardoso, Ana M. Rosa da Costa, Ana Grenha

**Affiliations:** 1 CBMR—Centre for Biomedical Research, University of Algarve, Faculty of Sciences and Technology, Campus de Gambelas, Faro 8005-139, Portugal; E-Mails: susananasus@gmail.com (S.R.); lurdes_cardoso17@sapo.pt (L.C.); 2 CIQA—Algarve Chemistry Research Centre and Department of Chemistry and Pharmacy, Faculty of Sciences and Technology, University of Algarve, Campus de Gambelas, Faro 8005-139, Portugal; E-Mail: amcosta@ualg.pt

**Keywords:** biocompatibility, chitosan, chondroitin sulfate, nasal delivery, polyelectrolyte complexes, polysaccharides, protein delivery, pulmonary delivery

## Abstract

Chitosan (CS) and chondroitin sulfate (CHS) are natural polymers with demonstrated applicability in drug delivery, while nanoparticles are one of the most explored carriers for transmucosal delivery of biopharmaceuticals. In this work we have prepared CS/CHS nanoparticles and associated for the first time the therapeutic protein insulin. Fluorescein isothiocyanate bovine serum albumin (FITC-BSA) was also used to enable comparison of behaviors regarding differences in molecular weight (5.7 kDa *versus* 67 kDa). Nanoparticles of approximately 200 nm and positive zeta potential around +20 mV were obtained. These parameters remained stable for up to 1 month at 4 °C. Proteins were associated with efficiencies of more than 50%. The release of FITC-BSA in PBS pH 7.4 was more sustained (50% in 24 h) than that of insulin (85% in 24 h). The biocompatibility of nanoparticles was tested in Calu-3 and A549 cells by means of three different assays. The metabolic assay MTT, the determination of lactate dehydrogenase release, and the quantification of the inflammatory response generated by cell exposure to nanoparticles have indicated an absence of overt toxicity. Overall, the results suggest good indications on the application of CS/CHS nanoparticles in respiratory transmucosal protein delivery, but the set of assays should be widened to clarify obtained results.

## 1. Introduction

Polymeric nanoparticles are one of the most explored carriers for mucosal and transmucosal delivery of biopharmaceuticals. This is mainly due to the high surface-to-volume ratio that improves both the drug loading capacity and the epithelial contact. Additionally, nanoparticulate systems might provide drug protection from aggressive environmental conditions [1,2] and potentiate increased drug absorption by reducing epithelial resistance to transport [3,4]. Natural polymers such as chitosan (CS) and chondroitin sulfate (CHS) have been frequently used as matrix materials in drug delivery systems. One of the reasons supporting their wide application is the usual compliance with requisites of biocompatibility and biodegradability [5]. However, they present additional advantageous characteristics. CS is a very well-known and characterized polysaccharide with reported mucoadhesive and absorption enhancement properties [6,7]. CHS is a physiological sulfated glycosaminoglycan existing in the normal lung and other body structures [8,9]. These polysaccharides have been reported to assemble into nanoparticles by a mild method of polyelectrolyte complexation [10,11], where the positively-charged amino groups of CS interact with the negatively-charged sulfate and carboxylate groups of CHS [1]. As a matter of fact, CS/CHS nanoparticles were reported in several works with varied applications, mainly including tissue engineering [12,13,14,15,16]. Other applications like cancer therapy [17,18], anticoagulant activity [10], anti-leishmanial activity [19] and cell transfection [20] were also referred. One sole research group reported their use in mucosal protein delivery [11,21], focusing an application in oral administration and using fluorescein isothiocyanate bovine serum albumin (FITC-BSA) as model protein. No report exists addressing the delivery of a therapeutic protein. The fact that CHS is endogenous of the lung raises the interest of this polysaccharide for pulmonary and, generally, respiratory drug delivery applications. However, contrary to CS, CHS was never reported in nanoparticle applications related with either lung or nasal protein delivery.

Delivering biomacromolecules is a challenge because of physicochemical properties that limit their absorption. The difficulty in using the oral route for their delivery, because of the high enzymatic content, has turned the attention to alternative routes of administration, like the nasal and the pulmonary. The ability of CS to adhere to mucosal surfaces and transiently open epithelial cell tight junctions, together with the endogenous character of CHS, potentiate the interest of CS/CHS nanoparticles for nasal or pulmonary transmucosal delivery of biomacromolecules. Nasal delivery of nanoparticles is possible by the direct administration of a suspension. Nevertheless, pulmonary delivery of these tiny carriers is generally hindered by their low mass, which causes predominant losses by exhalation. In this regard, a strategy to enable their delivery is necessary, and one possibility is the microencapsulation of nanoparticles, as was previously proposed by the authors [22,23]. With this approach, nanoparticles would be endowed with the required aerodynamic properties to reach the alveolar zone, where systemic absorption of drugs will take place.

In this study we aimed to verify for the first time the capacity of CS/CHS nanoparticles to associate and release the therapeutic protein insulin. FITC-BSA was also associated to the nanoparticles to enable the comparison of behaviors with respect to different molecular weights. Additionally, the biocompatibility profile of these nanoparticles was particularly focused regarding an application in respiratory delivery, evaluating their effect on the metabolic activity, membrane integrity, and inflammatory response of Calu-3 and A549 cells, two respiratory cell lines.

## 2. Experimental Section

### 2.1. Materials

CS in the form of hydrochloride salt (Protasan^®^ UP Cl 113, deacetylation degree = 75%–90%, molecular weight < 200 kDa), was purchased from Pronova Biopolymer (Sandvika, Norway). CHS, human insulin, FITC-BSA, phosphotungstate dibasic hydrate, glycerol, phosphate buffered saline (PBS) pH 7.4 tablets, Dulbecco’s modified Eagle’s medium (DMEM), penicillin/streptomycin (10,000 units/mL, 10,000 µg/mL), non-essential amino acids, l-glutamine 200 mM, trypsin-EDTA solution (2.5 g/L trypsin, 0.5 g/L EDTA), trypan blue solution (0.4%), thiazolyl blue tetrazolium bromide (MTT), lactate dehydrogenase (LDH) kit, sodium dodecyl sulfate (SDS), lipopolysacharide (LPS) and dimethyl sulfoxide (DMSO) were purchased from Sigma-Aldrich^®^ (Munich, Germany). Fetal bovine serum (FBS) was obtained from Gibco (Carlsbad, CA, USA). Human IL-6 and IL-8 Quantikine ELISA kits were from R&D Systems (Minneapolis, MN, USA). Ultrapure water (Mili-Q Plus, Milipore Iberica, Madrid, Spain) was used throughout. All other chemicals were reagent grade.

### 2.2. Cell Lines

The Calu-3 and A549 cell lines were obtained from the American Type Culture Collection (Rockville, MD, USA) and used between passages 35–45 and 20–30, respectively. Cell cultures were grown in 75 cm^2^ flasks in a humidified 5% CO_2_/95% atmospheric air incubator at 37 °C. For both cell lines, cell culture medium (CCM) was DMEM supplemented with 10% (v/v) FBS, 1% (v/v) non-essential amino acids solution, 1% (v/v) l-glutamine solution (200 mM) and 1% (v/v) penicillin/streptomycin. Medium was exchanged every 2–3 days and cells were subcultured weekly in the case of A549 cells and every 10 to 15 days for Calu-3 cell line.

### 2.3. Preparation of Chitosan/Chondroitin Sulfate Nanoparticles

Chitosan/chondroitin sulfate (CS/CHS) nanoparticles were prepared according to a previously described method of polyelectrolyte complexation between the amino groups of chitosan and both the sulfate and carboxylate groups of chondroitin sulfate [1]. Briefly, CS and CHS were dissolved in purified water to obtain stock solutions of 1 mg/mL (pH = 4.1) and 1.25 mg/mL (pH = 6.3), respectively. All solutions were filtered before use (0.2 µm filter, Whatman^®^, Dassel, Germany). The stock solution of CHS was then diluted to obtain the final desired concentrations. The amino groups present in CS have a typical pK_a_ around 6.2–7.0 [24], thus making the molecule positively charged after dissolution in water. In turn, CHS has a first pK_a_ value corresponding to sulfate groups at 1.5–2 and another one at 3–5 which corresponds to carboxylate groups [25]. Therefore, upon dissolution in water, CHS presents a negative charge. CS/CHS nanoparticles formed spontaneously upon the incorporation, under gentle stirring and at room temperature, of 0.8 mL of CHS of different concentrations (0.41–1.25 mg/mL) into 2 mL of a CS solution of 1 mg/mL, resulting in final theoretical CS/CHS mass ratios of 2/1–6/1.

Insulin (5.7 kDa; pI 5.3) and BSA (67 kDa; pI 4.7), in the form of FITC-BSA, were used as model proteins and associated to the formulation CS/CHS = 3/1. Insulin was dissolved in NaOH 0.01 M, while FITC-BSA was dissolved in ultrapure water (0.9 mg insulin or FITC-BSA in 0.6 mL of solvent). The protein solution was then incorporated into the CHS solution prior to nanoparticle formation. The amount of associated protein was 30% (w/w) calculated with respect to chitosan quantity in the formulation.

Nanoparticles were concentrated by centrifugation at 16,000× *g* on a 10 µL glycerol layer, for 30 min at 15 °C (Heraeus Fresco 17 centrifuge, Thermo Scientific^®^, Bremen, Germany). The supernatants were discarded and nanoparticles re-suspended in 200 µL of purified water.

### 2.4. Characterization of Chitosan/Chondroitin Sulfate Nanoparticles

#### 2.4.1. Morphological Analysis

The morphological analysis of CS/CHS nanoparticles was performed by TEM (Jeol-JEM^®^ 1011, Kyoto, Japan). Concentrated nanoparticle suspensions were obtained upon centrifugation, mounted on copper grids coated with a carbon film (Ted Pella^®^, Redding, CA, USA) and stained with a 2% (w/v) sodium phosphotungstate dibasic hydrate solution [26].

#### 2.4.2. Physicochemical Characterisation

The size and zeta potential of nanoparticles were measured by photon correlation spectroscopy and laser Doppler anemometry, respectively, using a Zetasizer^®^ NanoZS (Malvern Instruments, Malvern, UK). For the analysis of particle size and determination of electrophoretic mobility, each sample was diluted with ultrapure water and placed in an electrophoretic cell. Each analysis was performed at 25 °C (*n* = 3).

#### 2.4.3. Production Yield

The yield of nanoparticle production was determined by gravimetry. To do so, nanoparticles were prepared, isolated by centrifugation as described before, and the sediments were freeze-dried (Labconco^®^, Kansas City, MO, USA) over 24 h (*n* = 6). The production yield (PY) was calculated as follows:

(1) PY (%)=(Nanoparticle weight/Total solids weight) × 100

where *Nanoparticle weight* is the sediment weight after freeze-drying and *Total solids weight* is the total amount of solids added for nanoparticle formation.

#### 2.4.4. Surface Analysis by X-ray Photoelectron Spectroscopy (XPS)

The surface of CS/CHS nanoparticles was analyzed by XPS (K-Alpha, Thermo Scientific, UK) to determine their chemical composition. To do so, a droplet of nanoparticles (CS/CHS = 3/1, w/w) was placed directly on a polished monocrystalline silicon wafer, used as a sample holder. The droplet was then allowed to dry in a desiccator prior to the analyses. Solutions of the materials composing the matrix of the nanoparticles (CS and CHS) were analyzed separately as controls. The samples were exposed to an X-ray beam and the binding energies of characteristically-emitted photoelectrons were measured. This provided information on the elements from which they originate, as well as their chemical bonding. The measurements were carried out using monochromatic Al Kα X-ray source (*hv* = 1486.6 eV), and photoelectrons were collected from a take-off angle of 90° relative to the sample surface. The X-ray monochromatic spots were 400 µm in diameter and the corresponding sampling area was 0.1256 mm^2^. Measurements were performed in constant analyzer energy (CAE) mode with 100 eV pass energy for survey spectra and 20 eV pass energy for high-resolution spectra. Charge referencing was done by setting the lower binding energy C1s photopeak at 285.0 eV, the C1s hydrocarbon peak [27]. Surface elemental composition was determined using the standard Scofield photoemission cross section. Residual vacuum in the analysis chamber was maintained at around 6 × 10^−10^ mbar.

### 2.5. Determination of Protein Loading Capacity of Nanoparticles

The capacity of the nanoparticles (CS/CHS = 3/1, w/w) to associate the proteins was determined upon their separation from the aqueous preparation medium containing the non-associated protein by centrifugation (16,000× *g*, 30 min, 15 °C). The amount of free insulin was determined in the supernatant by the MicroBCA protein assay (Thermo Scientific™ Pierce™, Rockford, IL, USA), measuring the absorbances by spectrophotometry (Infinite M200, Tecan, Männedorf, Switzerland) at 562 nm. The amount of free FITC-BSA was obtained measuring directly the absorbance by spectrophotometry (Shimadzu UV-Vis Spectrophotometer UV-1700, Kyoto, Japan) at 494 nm. A calibration curve was made using the supernatant of unloaded nanoparticles in each case. Each formulation was assayed in triplicate (*n* = 3).

The protein association efficiency (AE) and the loading capacity (LC) of nanoparticles were calculated as follows:

(2) AE (%)=(Total protein amount−Free protein amount)/Total protein amount × 100

(3) LC (%)=(Total protein amount−Free protein amount)/Nanoparticle weight × 100

### 2.6. Determination of Protein Release Profile

The release of the proteins was determined by incubating a predetermined amount of the nanoparticles (CS/CHS = 3/1, w/w) in 12 mL of PBS pH 7.4 with horizontal shaking at 37 °C. At appropriate time intervals (0.5, 1, 2, and 4 h), individual samples were collected and filtered (0.22 µm filters Millex^®^-GV, low protein binding, Millipore, Madrid, Spain) and the amount of protein released evaluated in the supernatants by spectrophotometry, as described above, for each protein (*n* = 3).

### 2.7. Nanoparticle Stability Study

Aliquots of two CS/CHS nanoparticle formulations (3/1 and 4/1, w/w) were stored at 4 °C in the form of aqueous suspension. Size and zeta potential were monitored along time for up to one month, using the techniques described above (*n* ≥ 3).

### 2.8. In Vitro Biocompatibility Study

#### 2.8.1. Evaluation of Metabolic Activity

The *in vitro* cytotoxicity of CS/CHS nanoparticles (CS/CHS = 3/1, w/w), as well as that of the raw materials involved in nanoparticle production, was assessed in both Calu-3 and A549 cells by the metabolic assay thiazolyl blue tetrazolium bromide (MTT). A549 cells were seeded at a density of 1 × 10^4^ cells/well and Calu-3 cells at 2 × 10^4^ cells/well in 96-well plates, in 100 µL of the same medium used for culture in cell culture flasks. The cells were grown at 37 °C in a 5% CO_2_ atmosphere for 24 h before use [28]. Nanoparticle suspensions and solutions of the individual raw materials (CS and CHS) were evaluated separately for cytotoxicity. All samples were tested at three different concentrations (0.1, 0.5, and 1.0 mg/mL), over 3 and 24 h. Sodium dodecyl sulfate (SDS, 2%, w/v) was used as a positive control of cell death. Cell culture medium was used as negative control. Optimal cell seeding density and the absence of MTT conversion in the assay medium were confirmed in preliminary experiments. All formulations and controls were prepared as solution/suspensions in pre-warmed CCM without FBS immediately before application to the cells.

To initiate the assay, culture medium of cells at 24 h in culture was replaced by 100 µL of fresh medium without FBS containing the test samples or controls. After 3 or 24 h of cell incubation, samples/controls were removed and 30 µL of the MTT solution (0.5 mg/mL in PBS, pH 7.4) were added to each well. After 2 h, any generated formazan crystals were solubilized with 50 µL of DMSO. Upon complete solubilization of the crystals, the absorbance of each well was measured by spectrophotometry (Infinite M200, Tecan, Männedorf, Switzerland) at 540 nm and corrected for background absorbance (650 nm) [29].

The relative cell viability (%) was calculated as follows:

(4) Cell viability (%)=(A−S)/(CM−S) × 100

where A is the absorbance obtained for each of the concentrations of the test substance, S is the absorbance obtained for the 2% SDS and CM is the absorbance obtained for untreated cells (incubated with CCM). The latter reading was assumed to correspond to 100% cell viability. The assay was performed on three occasions with six replicates at each concentration of test substance in each instance.

#### 2.8.2. LDH Cytotoxicity Assay

A549 cells were seeded at a density of 1 × 10^4^ cells/well and Calu-3 cells at 2 × 10^4^ cells/well in 96-well plates, in 100 µL of the same medium used for culture in cell culture flasks. The cells were grown at 37 °C in a 5% CO_2_ atmosphere for 24 h before use [28]. After that time, culture medium was replaced by 100 µL of fresh medium without FBS containing CS/CHS nanoparticles (CS/CHS = 3/1, w/w) at the concentration of 1.0 mg/mL. The exposure was allowed for 24 h, after which the concentration of LDH present in the supernatant of the cells was determined. The quantification was performed using an appropriate kit and measuring the absorbance at 490 nm, with 655 nm as a reference wavelength. The incubation of cells with cell culture medium was used as a negative control of LDH release, assumed as the 100% release of the enzyme. Additionally, cells were also incubated with a lysis buffer provided by the manufacturer, which was used as positive control. The amount of released LDH (in percentage) was expressed as a function of that released by cells incubated with cell culture medium only. The assay was performed on three occasions with six replicates at each condition.

#### 2.8.3. Determination of Inflammatory Response

The inflammatory response generated by the exposure to nanoparticles was evaluated on Calu-3 cells. To perform the assay, the cells were seeded on 96-well plates (2 × 10^4^ cells/well) and, after 24 h incubation at 37 °C in 5% CO_2_ atmosphere, exposed to nanoparticles at a concentration of 1 mg/mL. The exposure to lypopolysacharide (LPS; 10 μg/mL) was used as positive control [30,31], while cells incubated with CCM were used as negative control. All formulations and controls were prepared as solutions/suspensions in pre-warmed CCM without FBS immediately before application to the cells. After 24 h of exposure, the cell supernatants were collected and centrifuged (10 min, 16,000× *g*, 15 °C, centrifuge 5804R, Eppendorf, Germany). The levels of IL-6 and IL-8 existing in Calu-3 cell supernatants were determined by quantitative ELISA using human IL-6 and IL-8 ELISA kits, according to the manufacturer’s instructions. The supernatants were appropriately diluted (approximately 10 times for IL-6 and 50 times for IL-8). The assay was performed on three occasions with two replicates of test substance in each instance. The concentration of individual cytokines for each sample is expressed as percentage of the negative control (incubation with CCM).

### 2.9. Statistical Analysis

The *t*-test and the one-way analysis of variance (ANOVA) with the pairwise multiple comparison procedures (Holm-Sidak method) were performed to compare two or multiple groups, respectively. All analyses were run using the SigmaStat^®^ statistical program (Version 3.5) and differences were considered to be significant at a level of *p* < 0.05.

## 3. Results and Discussion

### 3.1. Preparation and Characterisation of Nanoparticles

**Figure 1:**
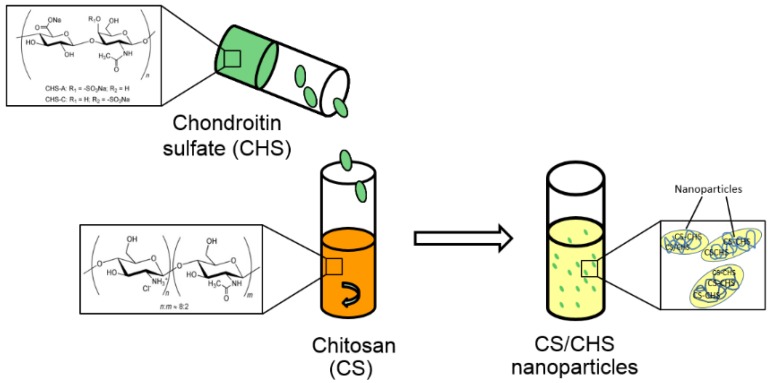
Representative scheme of the formation of CS/CHS nanoparticles by polyelectrolyte complexation.

Chitosan/chondroitin sulfate (CS/CHS) nanoparticles were successfully obtained by a process of polyelectrolyte complexation. Nanoparticle production by this method is mediated by the electrostatic interaction occurring between the negatively charged sulfate and carboxylate groups of chondroitin and the oppositely-charged amino groups of chitosan, as depicted in Figure 1. Polyelectrolyte complexation has been described to have advantages regarding the hydrophilic environment and the mild preparation conditions devoid of organic solvents or high shear forces [1,32]. Considering the pH of the involved solutions (4.1 for CS and 6.3 for CHS), the polymers display positive and negative charges, respectively. In the case of CS, a degree of deacetylation of 86%, to which corresponds 0.86 positive charges per monomer, and a *M*_w_ of 113 kDa, have been reported [33]. The mean molar mass for the monomer (198 g/mol) may be obtained by ponderation of the molar masses of the acetylated and deacetylated units. As for the CHS used in the present work, it was reported as being a mixture of 64% CHS-A and 27% CHS-C disaccharide units of _D_-glucuronic acid and monosulfated *N*-acetylgalactosamine, with a *M*_w_ of 21 kDa [34], these values being in line with those reported for CHS of the same origin (bovine cartilage), but from different suppliers [35]. In the former disaccharide unit, the sulfate ester is on position 4, while in the latter, it lies on position 6 [36]. The repeating unit in CHS has a mean molar mass of 494 g/mol, obtained by ponderation of the sulfated (91%) and unsulfated (9%) units, and 1.91 negative charges, one due to the carboxylate group and 0.91 from the sulfate group in sulfated units.

In the process of formation of CS/CHS nanoparticles, the concentration of chondroitin sulfate was varied in order to obtain different mass ratios, from 2/1 to 6/1. By dividing the charge of each repeating unit by its molar mass, a charge per mass ratio is obtained for each polymer. In a *n*/1 formulation of CS/CHS, the (+/−) charge ratio may be obtained as follows:

(5) (+/−) charge ratio =n . charge per mass (CS)/charge per mass (CHS)

Due to the similarity between the charge per mass ratios of both polymers (4.34 × 10^−3^ and 3.80 × 10^−3^ charges/g for CS and CHS, respectively), charge ratios do not significantly deviate from the corresponding mass ratios.

**Figure 2:**
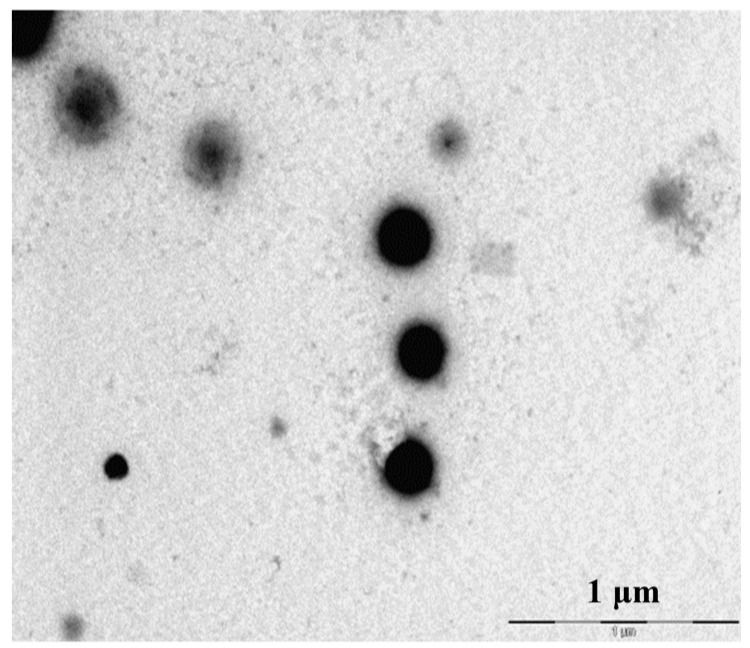
TEM microphotograph of representative CS/CHS nanoparticles (CS/CHS = 3/1, w/w).

Upon mixing the two polysaccharides in the referred range of mass ratios, a clear Tyndall effect was observed in all cases, indicating the formation of colloidal structures. As observed in the transmission electron microphotograph displayed in Figure 2, the morphology of CS/CHS nanoparticles corresponds to a compact structure with tendency to exhibit spherical shape, as has been described for many formulations of polysaccharide-based nanoparticles prepared by polyelectrolyte complexation [11,37,38,39,40].

As demonstrated in Table 1, the properties of unloaded nanoparticles did not vary significantly between the various mass ratios, with the exception of the size of formulation 6/1 (183 nm), which decreased significantly (*p* < 0.05), compared with the other formulations (between 206 and 219 nm). The absence of variations was not expected, as varying the amount of one of the polymers, and therefore the amount of charges, could result in different nanoparticle characteristics. However, similar observations were reported in other works of nanoparticle production involving different materials, by the same methodology [41]. There is evidence supporting that the process leading to polyelectrolyte complex formation is subdivided in the rapid formation of molecular or primary complex particles, followed by aggregation of primary particles to secondary particles, the former held together by long-range electrostatic interactions and the latter by short-range dispersive interactions [42,43]. Considering the polysaccharides’ molar masses, in both 6/1 and 5/1 formulations CS and CHS are present in approximately equimolar amounts. Approximately the same (reduced) number of primary complexes should form; however, in the former case, a more incomplete charge neutralization is expected (±charge ratio 6.9 and 5.7, respectively). This would result in enhanced electrostatic repulsion between primary particles, leading to low dispersive attraction and smaller particle sizes, as well as low yields. By increasing the amount of CHS, towards the 2/1 formulation, a larger number of primary particles form, which should tend to aggregate in larger particles as (±) charge ratios decrease (from 4.6 to 2.3). However, that was not the case, with all four formulations (5/1 to 2/1) presenting almost invariant particle sizes, which should mean that more particles formed; thus, the slight increase in yield observed from the 4/1 formulation on [43]. The fact that all formulations present similar surface potentials of ~19 mV, seems to corroborate this hypothesis, as this potential should correspond to a repulsive electrostatic force overcoming the dispersive interactions and therefore preventing the particles from growing further.

**Table 1:** Physicochemical characteristics and production yield of unloaded CS/CHS nanoparticles (mean ± SD, *n* ≥ 4).

CS/CHS (w/w)	Charge Ratio (±)	Size (nm)	ζ Potential (mV)	Production Yield (%)
2/1	2.3	216 ± 13	+21 ± 3	21 ± 3
3/1	3.4	212 ± 15	+19 ± 3	18 ± 8
4/1	4.6	219 ± 19	+17 ± 3	19 ± 3
5/1	5.7	206 ± 14	+18 ± 1	14 ± 2
6/1	6.9	183 ± 5	+18 ± 2	14 ± 6

CHS: chondroitin sulfate, CS: Chitosan, ζ: zeta.

Furthermore, strong variances in nanoparticle behavior due to size differences are not expected after administration, when comparing the five formulations, as the nominal difference is only of ~35 nm. Still, although not many reports are available in the literature to permit a comparison, the determined sizes are in line with those already reported. For instance, the formulation CS/CHS = 2/1 displays a size of 216 ± 13 nm which is approximate to that of 250 ± 6 nm found in another work for a mass ratio of 2/1.4 [11], although it is important to consider that the used polysaccharides might exhibit different properties.

The prepared nanoparticles display a positive zeta potential, coincident with the higher amount of chitosan that is present in all formulations. No statistical differences were found in the production yields, although a trend is observed regarding a progressive decrease with the incorporation of lower amounts of CHS.

The performance of subsequent tasks required the selection of one formulation. Nanoparticles CS/CHS = 2/1 were the first choice, essentially because they had the highest production yield, although this was not significantly different from others. However, as will be exposed in the corresponding section, the association of protein was not successful and, therefore, formulation CS/CHS = 3/1 was chosen.

Nanoparticles (CS/CHS = 3/1, w/w) were further analyzed by XPS to chemically characterize their surface and evaluate the distribution of both polymers in the carriers. Table 2 displays the percentage of each chemical element present in the sample of either the raw materials individually (CS and CHS), used as controls, or nanoparticles.

**Table 2:** Surface elemental composition (atomic %), determined by XPS, of chitosan (CS), chondroitin sulfate (CHS), and chitosan/chondroitin sulfate nanoparticles (CS/CHS NP).

Element	CS (%)	CHS (%)	CS/CHS NP (%)
C	59.71	52.29	56.18
O	30.53	34.86	36.68
N	5.75	4.27	5.46
S	0	2.84	1.68
Cl	4.01	0.35	0
Na	0	5.38	0
Ratio C/N	10.38	12.24	10.29
Ratio N/S	–	1.50	3.25
Ratio C/O	1.96	1.50	1.53

The final chemical composition of a sample can be obtained from core photoemission intensity peak areas using the Shirley background subtraction technique from the survey spectra. The element composition can be quantified by using X-ray photoelectron intensity values and the Scofield theoretically-derived set of atomic sensitivity factors. Some of the samples showed an intensive silicon (Si) signal (data not shown), which is attributed to the substrate, as a consequence of an incomplete coating of the substrate surface with the sample. These Si signals do not compromise the obtained results and are not included in percentages shown in Table 2.

The analysis of the individual raw materials detected the expected elements, such as carbon (C), oxygen (O), nitrogen (N) and, for chondroitin sulfate, sulfur (S). Apart from the typical C, O, and N, chitosan composition revealed the presence of Cl (4.01%), because the used chitosan is a hydrochloride salt. Similarly, Na was detected in CHS, as the polymer is a sodium salt.

The obtained CS composition (59.71% C, 30.53% O and 5.75% N) is very approximate to that found in other XPS analyses of chitosan [37,44]. The existing differences are attributed to the fact that different forms of CS were used: the free amine in the former studies and the hydrochloride salt in the present one. The different counter-ion (acetate, since neutral CS is usually dissolved in acetic acid solutions, and chloride, respectively) may lead to differences in the conformation of the polysaccharide chain, thus affecting the groups more exposed to the surface. Moreover, in the former case, the presence of acetate ions would contribute to the C, H, and O percentages, leading to values different from the ones obtained in this case. In turn, no data was found on the literature regarding the elemental composition of CHS expressed in atomic percentages, hindering any possible comparison. The atomic percentages determined for CS/CHS nanoparticles were 56.18% C, 36.68% O, 5.46% N, and 1.68% S, the content of S being necessarily attributed to the presence of CHS and meaning that the nanoparticle surface has a contribution of both polymers, as was expected. This possibly indicates that both polymers have an even distribution through the whole nanoparticle structure. Additionally, the percentage of N detected for nanoparticles (5.46%) is more similar to that of chitosan (5.75%) than chondroitin sulfate (4.27%), which complies with the higher amount of chitosan that is present, as indicated by the theoretical mass ratio of 3/1. The percentages of C, O, and N were comparable to those reported in other works for the analysis of chitosan-based nanoparticles, either composed of chitosan/tripolyphosphate (CS/TPP) [45,46] or chitosan/carrageenan/tripolyphosphate (CS/CRG/TPP) [37]. The slight variations that are registered are explained by the use of different polymers to complex chitosan in the production of nanoparticles, apart from the application of varied mass ratios. To date, a very limited number of nanoparticle reports show an analysis using this technique, making the establishment of comparisons a difficult task. Additionally, there are different possibilities to show the results and the atomic percentage is not always available.

### 3.2. Nanoparticle Stability Study

One of the most common problems of colloidal particles relies on their tendency for flocculation [47]. This effect is, in many cases, accompanied by a decrease in zeta potential to values below 30 mV, either positive or negative, where the repulsive forces are not enough to maintain nanoparticles separated from each other, leading to aggregation. Producing systems that maintain their physicochemical parameters stable a long time is, therefore, a major challenge and an urgent need.

Taking this into account, aqueous suspensions of CS/CHS nanoparticles (CS/CHS = 3/1, w/w) were monitored for their storage stability along time, concerning size and zeta potential, being maintained at 4 °C for up to one month. This permits obtaining information on nanoparticle stability in the re-suspension medium, which is relevant for instance when nanoparticles are an intermediate product of another drug delivery system, as reported in some works of our group [46,48]. Nanoparticles CS/CHS = 4/1 were also used in this study to enable a comparison of behaviors and disclose any effect that could be due to the presence of a higher amount of one of the polymers.

**Figure 3:**
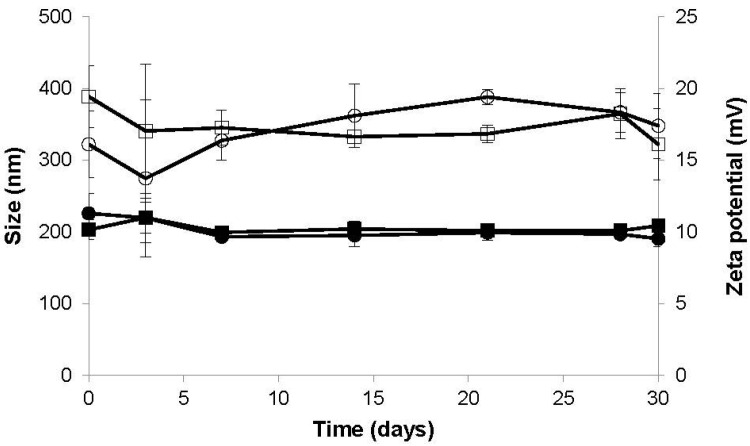
Evolution of the size (dark symbols) and zeta potential (empty symbols) of CS/CHS = 3/1 (circles) and CS/CHS = 4/1 (squares) nanoparticles as a function of time, upon storage of the aqueous suspension of nanoparticles at 4 °C (mean ± SD, *n* = 3).

As can be observed in Figure 3, both formulations of nanoparticles registered stability in aqueous suspension when stored at 4 °C, for a period of 30 days. No statistically significant changes were observed on nanoparticle size or zeta potential. It is further indicated that the stability is not apparently dependent on the mass ratio of the polymers, as both tested formulations exhibit a similar stable behavior.

Other polysaccharide-based nanoparticles produced by our team were previously reported to keep constant physicochemical properties for a similar time span in the same storage conditions. This was the case for nanoparticles composed of (1) chitosan, carrageenan and tripolyphosphate [37], (2) chitosan and sulfated pullulan, and (3) aminated pullulan and carrageenan [49]. Considering the results obtained in the present stability study, it can be said that CS/CHS nanoparticles remain physicochemically stable for a period that potentiates their application as drug delivery systems.

### 3.3. Association of Model Proteins

After the initial development and characterization of CS/CHS nanoparticles, the formulation CS/CHS = 2/1 was selected as first option to associate model proteins, as referred before. However, the attempt was not successful, as precipitation took place upon addition of the macromolecules. This was attributed to the presence of an excess of negative charges to neutralize the positive amino groups of chitosan (both from CHS and proteins), not permitting the formation of nanostructures [22,50]. Therefore, the formulation 3/1 was the following choice.

Insulin and FITC-BSA were used as model proteins, their selection being based on the different molecular weight (5.7 kDa and 67 kDa, respectively), which permits establishing a comparison between proteins of different characteristics. Additionally, the fact that they are very frequently used as models in drug delivery facilitates any comparison to be established with other works. As can be observed when comparing data from Table 1 and Table 3, no statistically significant differences were found in nanoparticle size upon the association of the proteins, but there was a significant increase in the production yield from 18% to 26%–29% (*p* < 0.05). The latter is justified by the proper mechanism of production of nanoparticles. The inclusion of the proteins, which were negatively charged at the working pH, provides an extra amount of negative charges, which interact with the positively-charged amino groups of chitosan, leading to an increase in the number of produced nanoparticles [51].

**Table 3:** Physicochemical characteristics, production yield, association efficiency and loading capacity of insulin- and FITC-BSA-loaded CS/CHS (3/1, w/w) nanoparticles (mean ± SD, *n* ≥ 4).

Protein	Size (nm)	ζ Potential (mV)	Production Yield (%)	Association Efficiency (%)	Loading Capacity (%)
Insulin	240 ± 44	+40 ± 2	29 ± 7	50.1 ± 3.6	36.5 ± 2.6
FITC-BSA	239 ± 4	+17 ± 1	26 ± 5	59.9 ± 4.5	40.9 ± 3.1

FITC-BSA: fluorescein isothyocyanate labelled bovine serum albumin, ζ: zeta.

Curiously, while the association of insulin resulted in a strong increase in the zeta potential, from +19 mV to +40 mV, that of FITC-BSA did not induce a significant alteration in this parameter. The latter effect was also observed by us in a previous work [49]. Insulin presents a dipolar charge anisotropy, which leads to the formation of aggregates of several protein molecules both under physiological conditions and at room temperature, low ionic strength and pH near pI, by the electrostatic attraction of the positive domain of one molecule and the negative “patch” of a neighboring one (the so-called “isoelectric precipitation” of insulin) [52]. It is possible that, by a similar process, free insulin molecules adsorb to the surface of the recently-formed, positively-charged, nanoparticles in a higher extent than BSA does. This would account for both the higher zeta-potential of insulin-loaded nanoparticles and the more pronounced burst release observed for this protein.

Both proteins were effectively associated to the nanoparticles, with encapsulation efficiencies of 50%–60%, which resulted in loading capacities of 37%–41%. This means that, in spite of the different molecular weight of the tested proteins, this does not translate into different abilities of the nanoparticles to associate the proteins. Another work available in the literature reported an encapsulation of 90% FITC-BSA in CS/CHS nanoparticles, but a different chitosan was possibly used [21]. While in the present work the deacetylation degree of chitosan is 75%–90% (provided by supplier), in the mentioned work it was determined to be 90.2%. If the chitosan used in the present work does not have 90%, the existing difference will mean a lower amount of charged amino groups available to associate the protein, thus resulting in lower association efficiency.

### 3.4. In Vitro Protein Release Study

**Figure 4:**
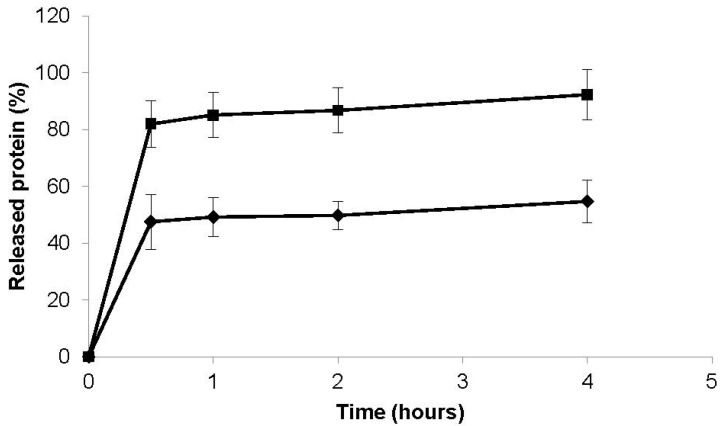
Release profiles of insulin (■) and FITC-BSA (♦) from CS/CHS nanoparticles (CS/CHS = 3:1, w/w) in PBS pH 7.4 at 37 °C (mean ± SD, *n* ≥ 3).

Figure 4 depicts the release profile evidenced by both proteins upon encapsulation in CS/CHS nanoparticles. The assay was conducted in PBS pH 7.4 as this medium is widely used as physiological medium, but it further mimics the lung lining fluid regarding the pH of lung environment, which is approximately 7 [53,54]. As can be observed in the figure, the release pattern is actually similar for both proteins, corresponding to a biphasic release. This biphasic behavior is coincident with reports on the literature describing chitosan-based nanoparticles, performed with diverse nanoparticle compositions and independently of the associated proteins [38,40,45,55,56].

The usual justification is the probable positioning of a certain amount of protein closer to the surface of the nanoparticles, which releases faster [40,45]. The protein that is in more interim locations of the nanoparticles would thus take longer to contact with solvent, solubilize and be available for quantification. In the present work, that is probably the explanation for BSA, but for insulin another justification should be added. In fact, after insulin association the nanoparticle zeta potential registered an increase from +19 mV to +40 mV, which did not occur for BSA. This suggests a real encapsulation of insulin rather than a preferential location on the surface. The typical burst release of insulin is usually justified by weak interactions occurring between chitosan and insulin, which enables insulin release by a simple dissociation mechanism [38,40]. Possibly, the high percentage released in initial moments in this work is a cumulative effect from both the location of some protein in the surface and the weak binding to the polymeric matrix, with a stronger contribution of the latter. However, there is a marked difference between proteins regarding the released amounts along time. While insulin evidences a burst release of 80% in the first instants of the assay, completing the release in approximately 4 h, FITC-BSA only releases 50% in the same period. This difference has also been frequently referred in the bibliography. In fact, insulin is generally described to assume a burst or a very rapid release from chitosan-based nanoparticles independently of the release medium and, specifically, regardless of its pH [22,38,40,51]. Contrarily, BSA typically evidences a slow release. Calvo *et al*., described a release of 8%–30% from chitosan/tripolyphosphate nanoparticles upon 48 h in an aqueous solution of trehalose (5%, w/v), depending on the initial protein loading [45,55]. In a previous work of our group testing pullulan/chitosan nanoparticles, approximately 30% of BSA released in 4 h [49].

In a work reporting the production of FITC-BSA-loaded CS/CHS nanoparticles a somewhat different behavior was described. In that case, FITC-BSA registers a release of approximately 14% after 1 h and approximately 20% at 4 h, which is approximately the half of what we obtained. The authors have further shown a release of 70% in 90 h [21]. These variances are probably related with the use of chitosan of different characteristics, which leads to a different binding between chitosan and the protein.

The different release rate of both proteins is consistent through several works and is probably associated with different binding patterns between the polymers and each of the proteins, as was also suggested by the resulting zeta potential of the formulations.

### 3.5. Biocompatibility Study of CS/CHS Nanoparticles

The evaluation of the biocompatibility profile of formulations designed for drug delivery is an up-to-date subject, referred as mandatory for any formulation being proposed [57]. While an accurate determination of toxicity can only be determined *in vivo*, a variety of *in vitro* toxicological assays, performed in adequately selected cell lines, might provide important first indications [58,59]. The evaluation must to be contextualized with a specific route of administration and dose of the material [57]. Moreover, the raw materials used in carrier matrix and the carriers should be evaluated separately [57,60,61], as it is assumed that the proper carrier structure might affect the final toxicological behavior [57,60].

To address these issues in a comprehensive way, this study provides the evaluation of the carriers by three different assays, evaluating different aspects of cellular response: (1) metabolic activity (MTT assay), (2) membrane integrity (LDH release), and (3) inflammatory response (detection of IL-6 and IL-8). The assays were performed on Calu-3 and A549 cells, both representing the respiratory epithelia. More specifically, while A549 cells represent the alveolar zone, Calu-3 cells are bronchially-derived and are the most used model of respiratory epithelium as a whole, being also representative of the nasal epithelium.

#### 3.5.1. Evaluation of Metabolic Activity

**Figure 5:**
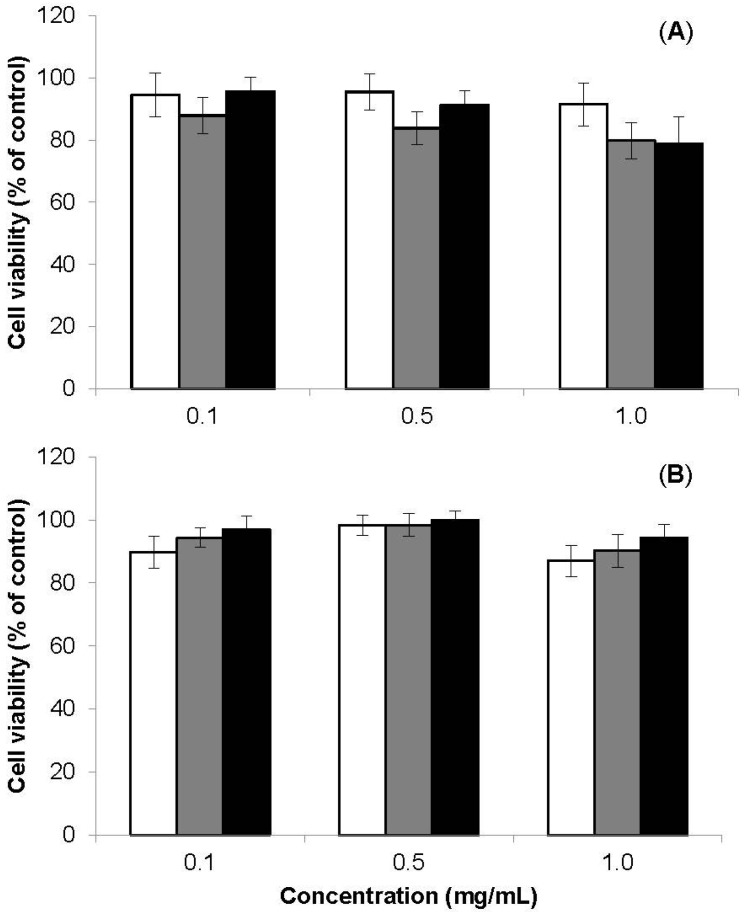
(**A**) A549 cells and (**B**) Calu-3 cells viability measured by the MTT assay after 24 h exposure to the raw materials chitosan (□) and chondroitin sulfate (■), and to CS/CHS nanoparticles (■; CS/CHS = 3/1, w/w). Data represent mean ± SEM (*n* = 3, six replicates per experiment at each concentration).

The cytotoxicity evaluation (MTT) was performed at both 3 h and 24 h. The exposure of both cell lines to the nanoparticles for 3 h did not result in a significant decrease of cell viability, all values being above 90% (data not shown). The prolongation of the exposure to 24 h revealed only a very slight decrease on cell viability (Figure 5), which is devoid of significance as the values remained close to 80%–90% in all cases. Therefore, no cytotoxic effect was considered to occur.

Other studies reported the absence of a toxic effect of chondroitin-based nanoparticles. One study of polylactide-chondroitin sulfate nanoparticles reported no toxicity in Caco-2 cells (cell viability > 100%) upon 3 days of incubation [62]. In another work, chondroitin sulfate was used to prepare nanoparticles and encapsulate selenium, thus strongly decreasing the toxicity of this molecule. The nanoparticles induced chondrocyte cell viability above 70% for concentrations up to 34 ng/mL [63].

Hu *et al*., also reported absence of toxicity of CS/CHS nanoparticles in Caco-2 cells upon incubation for 72 h, although the tested concentration was only 0.1 mg/mL [11,21]. Another work reported a similar observation in the same cell line, but in that case CS/CHS nanoparticles were incubated at concentrations up to 1 mg/mL, although only for 3 h [10]. A test in human adipose stem cells also revealed absence of toxicity at concentration of CS/CHS nanoparticles up to 2 mg/mL for an exposure time of up to 1 week [14]. When a chitosan derivative was used (chondroitin-carboxymethyl trimethylchitosan nanoparticles), cell viabilities of human dermal fibroblasts were above 80% for nanoparticle concentrations up to 1 mg/mL and an exposure of 24 h [13]. CS/CHS nanoparticles revealed an IC50 of 1189 μg/mL in murine macrophages [19]. Chitosan-pDNA complexes coated with chondroitin sulfate also induced a negligible decrease in COS-7 cell viability upon incubation for 24 h, although the concentration of nanoparticles is not disclosed [20]. As a whole, the data registered in this work, together with the observations available in the literature, indicate that nanoparticles based on chondroitin and chitosan do not cause overt toxicity in a wide range of cells.

#### 3.5.2. LDH Release

The release of LDH indicates the disruption of cell membrane, thus permitting the leaking of this cytoplasmic enzyme. The loss of intracellular LDH and its release to the culture medium is an indicator of irreversible cell death due to cell membrane damage [64,65]. The assay thus evaluates cell membrane integrity and complements the results obtained by the MTT assay.

**Figure 6:**
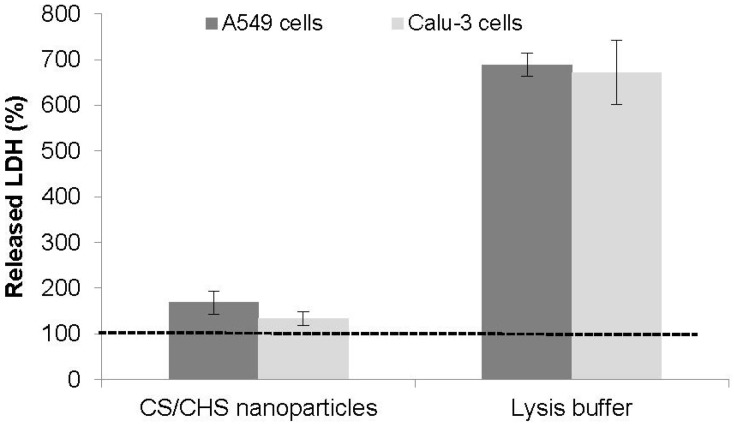
Effect of CS/CHS nanoparticles (CS/CHS = 3/1 (w/w), 1 mg/mL) on LDH release from Calu-3 and A549 cells upon 24 h exposure. Data represent mean ± SEM (*n* = 3, six replicates per experiment). Dotted line (100% LDH) represents the result of incubation with cell culture medium, used as control.

In this study, the amount of LDH released by A549 and Calu-3 cells exposed to CS/CHS nanoparticles was determined, using as control both the incubation with cell culture medium (negative control, assumed as 100%) and the exposure to a lysis buffer (positive control). The latter corresponds to the maximum amount of cytoplasmic enzyme that can be released, while the former is the minimum. As observed in Figure 6, the exposure of both cell lines to CS/CHS nanoparticles induced the release of an amount of LDH that is comparable to that of the negative control, as no statistically significant differences were detected. On the contrary and as expected, the treatment with the lysis buffer resulted in a very strong increase (*p* < 0.05) in the quantified LDH, because of the complete membrane disruption induced by the buffer. The results thus indicate an absence of cell membrane damage provoked by CS/CHS nanoparticles.

None of the works reporting the application of chitosan/chondroitin sulfate nanoparticles provides data on LDH release from cells upon exposure to the carriers. The same occurs for chondroitin sulfate-based nanoparticles but, on the contrary, chitosan-based nanoparticles were tested for this aspect in some occasions. Notwithstanding the different assay conditions in all works (cell line, time of incubation, concentration of nanoparticles, *etc.*), the general information to retain is that nanoparticles do not induce significant cellular damage when compared with cells incubated with the respective cell culture media [66,67,68,69].

Although the results of this assay indicate an absence of membrane damage upon contact with CS/CHS nanoparticles, this outcome should be taken cautiously. In fact, while no membrane injury is observed, the uptake of nanoparticles by epithelial cells mediated by a mechanism of macropinocytosis is described as a possibility [70]. In that case, which is particularly relevant for larger-size nanoparticles, no membrane damage is expected to occur and further studies would be needed to conclude on a cytotoxic profile.

#### 3.5.3. Inflammatory Response

Apart from determining the effect on cell viability induced by the exposure to drug carriers, evaluating the generation of a potential inflammatory response is a complementary assay towards the definition of a biocompatibility profile [30,71,72]. IL-6 and IL-8 are two cytokines suitable for this assessment, as IL-6 is responsible for neutrophil activation and IL-8 is a chemotactic agent for inflammatory cells [73]. The detection of these factors on cell supernatant should be indicative of an inflammatory effect.

**Figure 7:**
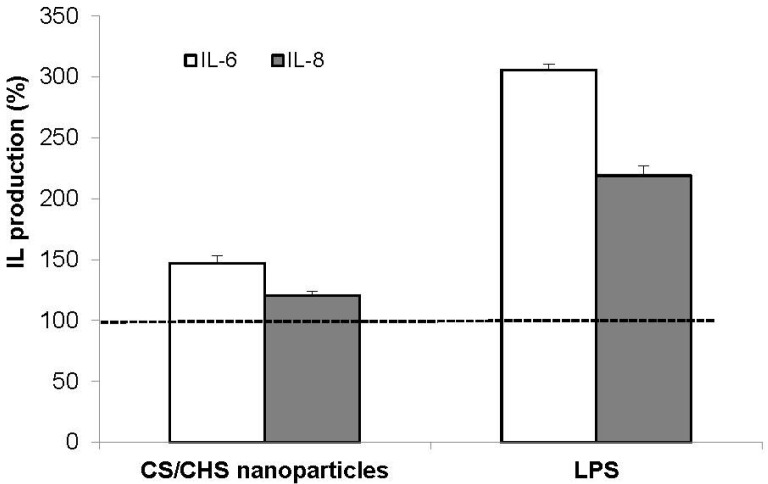
IL-6 and IL-8 secretion by Calu-3 cells exposed for 24 h to CS/CHS nanoparticles (CS/CHS = 3/1 (w/w), 1 mg/mL) and lipopolysaccharide (LPS, 10 μg/mL). Data represent mean ± SEM (*n* = 3). Dotted line (100% IL production) represents the result of incubation with cell culture medium, used as control.

In this assay, Calu-3 cells were exposed to CS/CHS nanoparticles (1 mg/mL) for 24 h and the induction of an inflammatory phenotype was determined by the quantification of released IL-6 and IL-8. The obtained results are depicted in Figure 7. LPS was used as a positive control (10 μg/mL) and cell culture medium as a negative control.

The amount of cytokines released in response to culture medium was considered 100% and all the other results are presented with respect to that value. As expected, a basal concentration of the two cytokines was detected in the supernatant of Calu-3 cells upon incubation with culture medium only [30,74]. Moreover, at this basal level, IL-8 was secreted to a higher concentration than IL-6 [30,75].

It is observed that the release of cytokines is slightly increased upon contact with the nanoparticles, an effect that is more visible for IL-6 (*p* < 0.05). In nominal terms, IL-6 increased 47%, while IL-8, 20%. Although the effect was much lower than that obtained upon incubation with LPS, which is known for its ability to generate inflammatory responses, a slight inflammatory response is suggested.

In an isolated manner it is difficult to establish the physiological meaning of this result. While MTT results indicate that CS/CHS nanoparticles do not affect cell metabolic activity and the determination of LDH release indicates no alteration of membrane integrity, the release of cytokines does not establish a clear safety profile regarding inflammatory processes. Therefore, although the overall analysis of the biocompatibility studies does not suggest overt toxicity on respiratory epithelial cells, it is considered necessary to perform more and different studies that might help to define the safety of the formulation. In any way, a definitive response would only be clearly established after *in vivo* administration.

## 4. Conclusions

In this work, polyelectrolyte complexes assembled with chitosan and chondroitin sulfate efficiently associated (encapsulation efficiencies up to 60%) two proteins of different molecular weights, insulin with 5.7 kDa and FITC-BSA with 67 kDa, the former reported for the first time in these carriers. The physicochemical characteristics of nanoparticles remained stable for 1 month when storage occurred at 4 °C. Insulin release was faster than that of FITC-BSA, evidencing different binding patterns between the polymers and the proteins. A biocompatibility study of the nanoparticles in respiratory cell lines (bronchial Calu-3 and alveolar A549) provided indications on the general suitability for an application in nasal or pulmonary transmucosal delivery. The MTT assay revealed cell viabilities above 80% for up to 24 h exposure at the concentration of 1 mg/mL in both cell lines, while LDH release induced by nanoparticles was similar to that of cells incubated with culture medium. The determination of released cytokines (IL-6 and IL-8) evidenced a slight increase upon contact with nanoparticles (up to 47%). Although the overall data suggest the applicability of chitosan/chondroitin sulfate nanoparticles as carriers for transmucosal respiratory delivery, more assays are needed in the ambit of biocompatibility to establish the real potential of their application and clarify the results obtained herein.
